# Experiences of Organizational Practices That Advance Women in Health Care Leadership

**DOI:** 10.1001/jamanetworkopen.2023.3532

**Published:** 2023-03-20

**Authors:** Mariam Mousa, Belinda Garth, Jacqueline A. Boyle, Kathleen Riach, Helena J. Teede

**Affiliations:** 1 Monash Centre for Health Research and Implementation, School of Public Health and Preventive Medicine, Monash University, Melbourne, Australia; 2 Epworth Healthcare, Richmond, Australia; 3 Rural Health, Faculty of Medicine, Nursing and Health Sciences, Monash University, Melbourne, Australia; 4 Health Systems and Equity, Eastern Health Clinical School, Melbourne, Australia; 5 Adam Smith Business School, University of Glasgow, Glasgow, Scotland; 6 Monash Partners Academic Health Science Centre, Melbourne, Australia; 7 Endocrine and Diabetes Units, Monash Health, Melbourne, Australia

## Abstract

**Importance:**

Women are underrepresented in health care leadership positions. Organizational practices and culture play a key role in mitigating this disparity.

**Objective:**

To explore the experiences of women in leadership roles and inform how health care organizations can support the advancement of women into leadership.

**Design, Setting, and Participants:**

This qualitative study used a constructivist grounded theory approach applied over a 1-year period (May 1, 2021, to May 31, 2022) in a large private health care network in Australia. Women were eligible if they had been in leadership positions for more than 5 years. Purposive and theoretical sampling guided recruitment of 28 women, representing medical, nursing, and allied health specialties. Interviews lasted 1 hour, producing 500 pages of transcripts for analysis.

**Main Outcomes and Measures:**

The primary outcome was a model of organizational practices and conditions that advance women in health care leadership, extrapolated from the collective experiences of women in leadership. Key elements pertained to organizational patterns of interaction and group norms and behaviors that contributed toward women’s experiences of career advancement.

**Results:**

Overall, 28 women (23 [82%] White; 3 [11%] Southeast Asian) participated in the study, 10 (36%) of whom were in nursing, 9 (32%) of whom were in allied health, and 9 (32%) of whom were in medical disciplines. Organizational practices that advance women in health care leadership were highly dependent on conducive organizational culture enhancing women’s credibility and capability as leaders. Four interrelated elements were identified that create the necessary conditions for an organizational culture to advance women in health care leadership, including (1) identifying and actively addressing systemic barriers, (2) challenging gendered assumptions and expectations of leadership behaviors, (3) providing mentorship to shape career opportunities, and (4) determining how these conditions all contribute toward raising women’s credibility to enable internalizing a leadership identity. For women, advancing to leadership involved organizations moving away from ad hoc, inconsistent applications of gender equity practices and generating supportive practices that reinforced a workforce culture of credibility, collaboration, and continuous improvement to support women.

**Conclusions and Relevance:**

In light of persisting inequity in health care leadership, women’s experiences were captured in this qualitative study to identify organizational practices that support their advancement. Insights into factors that influence efficacy of these practices, including building a supportive culture and mentoring, are discussed. This research informs a National Health and Medical Research Council initiative with international collaborators to support organizations in advancing women in health care leadership.

## Key Points

**Question:**

How do organizational practices and conditions work together to advance women in health care leadership from the experiences of women?

**Findings:**

In this qualitative study of 28 women in medical, nursing, and allied health specialty leadership, a model was developed explaining how organizational practices advance women in health care leadership. Elements of this model facilitate a conducive organizational culture for women’s career advancement.

**Meaning:**

These findings suggest that the health care sector benefits from organizational efforts to redress issues that affect women’s advancement into leadership.

## Teaser

This qualitative study focuses on the collective experiences of women as stakeholders to explore how organizational practices and conditions work together to advance women in health care leadership.

## Introduction

Women continue to be underrepresented in health care leadership, limiting leadership diversity.^1,2^ There is broad awareness of the need for greater equity in leadership to retain an invaluable workforce and reduce current attrition rates following the COVID-19 pandemic.^3,4,5^ Research also shows that greater gender diversity in health care leadership is a key step toward eliminating health care disparities and improving quality of care, leading to more equity in health and social justice and improved health care outcomes for women and children.^3,4,5,6^ This provides a strong imperative for prioritizing greater diversity and equity in leadership for the health care sector.^7,8,9,10,11,12^

Greater gender diversity in leadership has also been found to improve organizational performance, with positive effects reported across employee engagement, satisfaction, and retention.^13,14^ This works toward supporting the realization of the Quadruple Aim (care, health, cost, and meaning in work)^15^ and creating the conditions for the health care workforce to find meaning in their work.^15^ Valuing the workforce and optimizing health care outcomes is important to enhance engagement, commitment, and expectations toward the collective conduct of the workforce and in turn the culture of the organization.^16,17^ With persistent gender inequity in health care leadership, the complexity of barriers, and slow progress, there is a need to accelerate change to deliver improvements in workforce retention and to improve health care and health outcomes.

The number of women in leadership has increased marginally at entry and midcareer levels, with men consistently overrepresented in high-level decision-making roles.^18,19,20^ Increasing the pipeline of qualified women alone is inadequate to redress the seemingly intractable barriers and challenges toward equity.^5,16^ It is recognized that efforts must move beyond a reliance on individual women navigating organizational workplaces that do not meet their needs, to deliver organizational and systems change that enables women’s career advancement.^1,2^ Furthermore, research shows that organizations with the cultural and structural ability to attract, maintain, and develop talented women are better positioned to serve their communities.^2,3,21^ However, changing ingrained organizational culture is complex and mandating change is insufficient,^22,23^ requiring greater research and dedicated effort in these areas.^19,20,24^

A comprehensive systematic literature review and meta-synthesis has identified what organizational-level interventions advance women in health care leadership.^19,25^ Interventions fell into 5 categories: (1) organizational processes, (2) awareness and engagement, (3) mentoring and networks, (4) leadership development, and (5) organizational support tools.^19^ A secondary meta-ethnographic analysis identified how these interventions may work, highlighting factors that enable implementation at the organizational level.^24^ These included demonstrable leadership commitment and accountability toward women’s advancement; the importance of incorporating intervention fit with the workforce (ie, ensuring fit between an intervention and the implementing context, including women’s lived experiences as a critical precursor of implementation success); and the development of a conducive cultural climate ready to support change for gender equity as key to effectively and sustainably implementing evidence-based interventions.^21,22^

Herein, we aim to extend that body of knowledge and focus on the collective experiences of women as stakeholders to explore how organizational practices and conditions work together to advance women in health care leadership. In doing so, we develop a model that explains how these interconnected conditions help to build an organizational culture conducive to supporting and advancing women in health care leadership.

## Methods

### Study Design

In this qualitative study, we used a constructivist grounded theory method,^26,27^ as this approach is appropriate for exploring the underlying processes inherent to an area of inquiry,^28^ enabling us to understand how organizational practices and conditions affect career advancement as experienced by women.^29^ Ethical approval for this qualitative study was obtained from the Monash University Human Research Ethics Committee. Participants provided verbal consent at the beginning of their interview, which was audio and video recorded. The study followed the Consolidated Criteria for Reporting Qualitative Research (COREQ) reporting guideline.

### Context

Australia has a multipayer public health care system supplemented by an insurance-supported private health care system. This study was performed in collaboration with participants from one of Australia’s largest private health care networks who reflected on their broad career journey. The private network did not influence the interpretation or results of the study.

### Recruitment

Purposive sampling was used to identify information-rich data sources and intentionally select participants who would enable us to answer our research question.^30,31^ An initial purposive sample of women from across professional and clinical disciplines in entry-level and middle management, senior, executive, directorship, and board leadership roles for more than 5 years were identified by the organization. The organizational executive group and the People and Culture team provided access to the range of participants. Contact details of those who agreed to participate were shared with the first author (M.M.), who provided an information pack and invited participants to a virtual platform link. Consistent with a grounded theory approach, further participant recruitment was guided by theoretical sampling, which directed additional data collection to inform and develop theoretical categories.^29,30^ Final sample size was determined when theoretical saturation was reached whereby categories had been fully identified, explored, and sufficiently explained.^28^ Self-reported race and ethnicity data were collected during the interviews; categories included Greek or European ancestry, Southeast Asian, and White. This information was important to the study, as it provided some context to the experiences of the women interviewed, but also demonstrated how the sample was somewhat reflective of the race and ethnicity breakdown in Australian society.

### Data Collection and Analysis

Semistructured online interviews were conducted by the first author, who had no prior connection to participants. The interviews were performed during a 1-year period from May 1, 2021, to May 31, 2022, and lasted up to 1 hour with an interview guide (eTable 1 in Supplement 1). This guide was informed by evidence on organizational practices shown to have impact on women’s advancement into leadership^19^ and piloted with the multidisciplinary authorship team.^19,24^ All interview recordings were transcribed verbatim and deidentified. Data were stored securely and managed using Dedoose, version 9.0.62 (SocioCultural Research Consultants LLC).

Data collection and analysis were conducted iteratively, with constant comparative analysis throughout, beginning with the first transcript.^28,30^ Systematic coding involved independent transcript review by 2 investigators (M.M. and B.G.) and open coding of the first 5 transcripts. This was a form of analyst triangulation and opened up the data; data were categorized and assigned meaning without predetermined codes or categories.^28,32,33,34,35^ Axial coding then involved comparing initial codes to one another and sorting into categories and subcategories according to attributes of the data. Coding categories were constantly compared, examined, defined, and refined, generating increasingly more abstract concepts.^29,35^ Memos during and after interviews also captured key points and linked analytical interpretation, starting with data distillation into coding categories.^28^ Finally, theoretical coding involved lifting analysis to identify interrelated concepts and relationships between categories and the development of an interpretive model reflective of the data.^26,30^

### Reflexivity

Throughout the research process, we considered the influence of our roles on data collection, analysis, and interpretation. Investigators included a qualitative researcher and a woman with a racial minority background who led the study (M.M.); an experienced qualitative and primary health care researcher with expertise in grounded theory methodology (B.G.); an experienced academic obstetrician-gynecologist and researcher, with expertise working with members of Indigenous and marginalized communities (J.A.B.); an experienced endocrinologist and health care researcher with expertise in health care improvement and implementation (H.J.T.); and an experienced health care researcher with expertise on the intersections of aging, gender, and career trajectories (K.R.). Regular meetings among 3 investigators (M.M., B.G., and H.J.T.) enriched analysis and enhanced interpretive rigour.^31^

## Results

Twenty-eight women occupying positions ranging from entry-level management to senior-level, executive directorship and board membership, participated across nursing (10 [36%]), allied health (9 [32%]), and medical (9 [32%]) disciplines. Overall, 2 participants (7%) were Greek or of European ancestry, 3 (11%) were Southeast Asian, and 23 (82%) were White. A model of organizational practices and conditions that advance women in health care leadership was generated, drawn from the collective experiences of women in leadership. Illustrative quotations are provided in the Table.

**Table.** Categories, Subcategories, and Exemplar Quotations

Example	Exemplar quotation (participant)
**Identifying and actively addressing systemic barriers**	
Organizational attention to barriers specific to women’s needs, targeting support	“Something that does worry me…how can I one day maintain this job, which I really want to, if I become a mother, and what are my options? I don’t know how comfortable I would feel…having that conversation of what I’m entitled to and what my options would be.” (PMD)
Providing equitable access for all individuals including to family responsibilities and ingrained societal expectations	“It’s interesting actually because I don’t have children. I think sometimes for women who are in leadership positions and don’t have children…there’s more of an expectation where you don’t have to leave at three o’clock…whereas women in positions that have children, may need to have that work-life balance, so I don’t know if we’re overtly supportive of that work-life balance in leadership particularly.” (PN)
Targeting women proactively with opportunities and support	“When you see potential in women, I think it’s really important that we recognize talent no matter where they are, communicate that talent, break down the stereotypes. I think having that awareness in our culture is really important.” (PAH)
Career opportunities—visibility and clarity	“There could be a really easy way for a hospital to do an audit of its practices and see—we should have someone this woman can talk to who looks like them…and be willing to be accountable to the results of that, and say, well, we clearly don’t have enough representation. What are we doing that stop women applying?” (PMD)
Career opportunities—the need to recognize and target women proactively	“When I applied for a role there, I was hoping for a leadership role, but I thought, you know, I probably need to start again…. When they offered me a leading role here, I was like, oh, my God, I’m going to grab when I was at ----, and here, the way they look after their leaders here, it’s just really different. It’s totally different.” (PN)
Credible and implementable policies and practices	“I think we need better promotion practices, not modules at the start of the year on workplace equity that no one takes any notice of.” (PMD)
	“If you are reported for saying racist, sexist…you will be called in for professional misconduct…. It’s going to be taken very, very seriously. I think unless people are explicitly told that from a very senior person within a hospital, they assume they can get away with it.” (PMD)
**Challenging gendered assumptions and expectations of leadership behaviors**	
Social and cultural cues around interactions as leaders	“I think women are disempowered because of gender…because we’re often seen as the more emotive, driven by our hearts…. Sometimes to be successful, you’ve got to be seen to be powerful and strong, tell people what they need to do…. I don’t think if there was a male in this role that they would have the same reaction.” (PN)
Perceived dichotomy between behavioral benchmarks for men and women	“I’ve been quite assertive in a meeting, but it’s been seen as aggressive, because a man can be aggressive, and it’s not seen as aggressive. It’s really interesting.” (PMD)
Descriptive and prescriptive organizational practices—important for counteracting gendered behavioral benchmarks	“I think if you are part of an organization, even if you’re in a relative position of power, and that organization starts bringing in these changes (ie, practices to support women) and saying, this is now what we stand for, this is what we’re doing. Like it or lump it. If you don’t like it, you don’t have to work for us, but this is what we’re doing. Here’s the evidence. This is what we know will happen. We’ll get better patient outcomes. Economic productivity improves. Everything is better in terms of workplace culture.” (PMD)
	“The people who are really resistant to change, and really hate it, and don’t want to see diversity in terms of power. If they leave, great, you’ve improved culture already, regardless. In terms of everyone else, you’ve made a visible display and a structural display of commitment and, hopefully, that emboldens women and other people from diverse backgrounds…to go, okay, I feel safer and I feel more confident. They can keep making change. The little steps that keep bringing women up and keep bringing from diverse backgrounds up. It’s like an escalator, you just keeping doing little steps at a time, and eventually it rolls over into a better environment.” (PAH)
**Mentorship as shaping experiences of career opportunities**	
Opportunities for the individual and the organization from being mentored	“I think it’s really all about opportunity. Having opportunities to learn from others. You tend to learn a lot from those around you that you don’t want to emulate, unfortunately. But it would be nice to be able to have access to some of those really strong leaders in the organization that you do want to emulate because of what you see and what you hear of them and from them. Some way to foster that culture of learning from each other.” (PAH)
Structure around mentoring needed to improve effectiveness of opportunities (ie, access)	“I think that’s at the goodwill of individuals, and the luck you have finding that mentor who’s willing to spend the time. There’s nothing formal to help engage that or there’s no requirement to do it. In fact, leaders should have mentors, really. Where are they learning from? How are they developing, besides being in the trenches every day?” (PN)
Organizational mentoring strategies increase commitment and advocacy of individuals	“You have all your leaders learning similar things and similar approaches that, obviously, would align with the organization’s ideology…overall concepts of this is the sort of approach we expect our leaders to take, that are open, transparent, whatever approachable. As opposed to leaders finding their own way, and taking their own approach. I think the organization would get a more consistent group of leaders and benefit from a more consistent approach.” (PAH)
Mentoring for generating better leaders	“Mentoring is really important for consistent leadership. I think the stronger your leadership team, the stronger the trust with your floor and your employees. You can’t do anything in health care without good employees. Surgeons in private health care won’t operate at your hospital unless they’ve got good patient outcomes. So, it’s a cycle of continuous improvement. Good staff that trust good leaders, they enforce good staff, that keep and retain good staff, means good patient care, means more surgeons want to come work for your hospital.” (PMD)
**Raising women’s credibility to enable internalizing a leadership identity**	
Navigating, overcoming, or correcting how women are perceived as leaders	“Whether that’s normally the traits and attributes you’d put towards a woman…. Maybe that’s not what people perceive a woman to be. But women in leadership have to be all of those things at times.” (PAH)
	“I think having women in leadership needs to be balanced by getting the right people for the right role at the right time…there is a risk of not getting the balance right if the right person isn’t in the role, and that causes more damage than good.” (PN)
Organizational embodiment of ideal leadership reinforcing traditionally masculine ways of leading, as the only credible way of leading	“It’s definitely about the perception of the power at the top of the tree, and what that top of tree looks like, and perhaps the perception about what it takes to be at the top, and whether that’s normally the traits and attributes you’d put towards a woman…women in leadership have to be all of those things at times.” (PAH)
	“One thing I learnt on the job back then, ‘it’ (ie, proving credibility) was a lot about self-resourcing and being assertive to find the connections within the organization to support your decisions.” (PMD)
	“It’s just being strategic about okay, how do I make this person not feel uncomfortable around me, so that they can give me what I need?” (PN)

Abbreviations: PAH, participant in allied health discipline; PMD, participant in medicine discipline; PN, participant in nursing discipline.

### A Model of Organizational Practices That Advance Women in Leadership

This model conceptually represents the path from workforce entry to leadership attainment as highly dependent on conducive organizational culture enhancing women’s credibility and capability as leaders. Four interrelated elements were identified that create the necessary conditions for an organizational culture to advance women in health care leadership, including (1) identifying and actively addressing systemic barriers; (2) challenging gendered assumptions and expectations of leadership behaviors; (3) providing mentorship to shape career opportunities; and (4) determining how these all contribute toward raising women’s credibility to enable internalizing a leadership identity. Each element will be presented in turn, followed by further examination on their interconnectedness (Figure).

**Figure.**
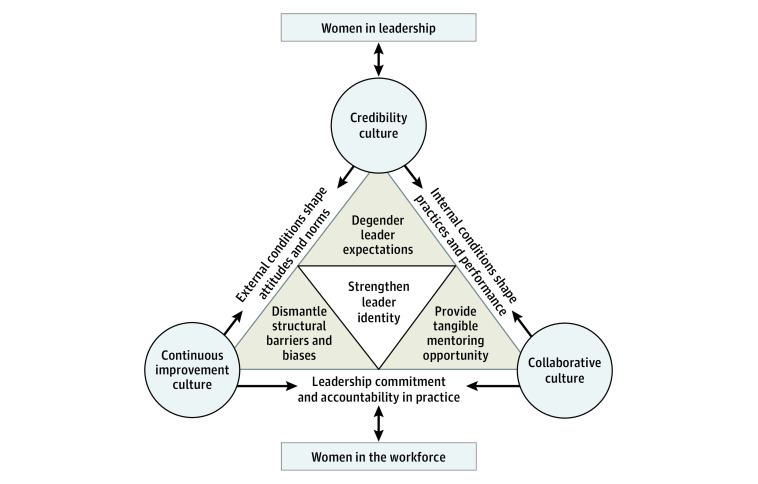
Conceptual Model for Advancing Women in Health Care Leadership Visual representation of how organizations can implement organizational practices that improve outcomes for women and their career advancement in the workforce.

#### Identifying and Actively Addressing Systemic Barriers

Gender equity at the organizational level was perceived by participants as the removal of systemic barriers and biases, enabling all individuals, including women, to have equal opportunity, specifically around access to benefits and/or resources that fit with needs and circumstances. Lack of a supportive organizational culture perpetuated inconsistent gender equity practices and undermined the provision of opportunities for women’s advancement and promotion. Participants conveyed a need for those who engage in the work ecosystem to develop a robust understanding of the systemic barriers faced by women, to implement meaningful practices to address them (eTable 2, subtheme 1.1, in Supplement 1). Participants consistently articulated barriers around family responsibilities and ingrained societal expectations, highlighting the need for all individuals to have equitable access to flexible work options not just related to caring responsibilities (eTable 2, subthemes 1.2 and 1.3, in Supplement 1).

Career opportunities were perceived as different for women and men, particularly around obtaining support and the need for visibility and clarity around opportunities and for proactive recognition and targeting of women (eTable 2, subthemes 1.4 and 1.5, in Supplement 1). Ensuring policies and practices balance idealistic thinking and realistic enactment was also important, by being credible and implementable and implemented, as opposed to a tokenistic equity rhetoric (eTable 2, subtheme 1.6, in Supplement 1). Overall, the experiences of women suggested an inclusive organizational culture is one that considers the barriers and enablers impacting women in the workplace and the context in which practical and procedural organizational rules operate, specifically around recruitment and promotion. Inconsistent, siloed, tokenistic, or absent gender-focused practices stymied progress; these were considered in need of recognition and dismantling.

#### Challenging Gendered Assumptions and Expectations of Leadership Behaviors

Social and cultural cues around women’s interactions as leaders were influential in career progression (eTable 2, subtheme 2.1, in Supplement 1). Notions pertaining to gender were articulated from traditional societal views concentrating men in agentic roles and relegating women to communal roles and responsibilities. Organizations were perceived to have a responsibility toward challenging and decentering how leadership is situated, eliminating the need for individuals to negotiate socially constituted gendered roles related to leadership. This reflected a perceived dichotomy between the expectation of women to meet different behavioral benchmarks compared with men, while simultaneously setting these behavioral benchmarks as different from those of a successful leader (eTable 2, subtheme 2.2, in Supplement 1). Having to yield to gender-congruent behaviors, avoid resistance, and fulfil expectations of others was perceived to further compromise the credibility of women in leadership roles.

Organizational practices in their descriptive and prescriptive forms were important for counteracting this effect and optimizing opportunities for all. Altering perceptions on what constitutes success included challenging an assumed objective nature to leadership and embracing both communal and/or agentic characteristics that are responsive to others’ needs and express agency, respectively (eTable 2, subtheme 2.3, in Supplement 1). Overall, seamless changes in perceptions, practices, and ultimately culture were recognized as unlikely. Incremental changes in organizational practices that cumulatively challenge internal cultural norms were considered likely to generate friction and resistance, seen as a necessary evil and an indication of true change.

#### Providing Mentorship to Shape Career Opportunities

Mentoring was considered the greatest contributor to career success, providing benefits to the mentor, mentee, and organization. Mentoring enabled information sharing and learning and offered opportunities for exposure to talented individuals, increasing productivity while enhancing and retaining important skill sets within the organization. Particularly effective mentoring included ready access to mentors and organizationally aligned mentoring opportunities; less effective mentoring involved limited access through lack of structure around mentoring opportunities that were largely left to chance (eTable 2, subtheme 3.1, in Supplement 1).

Organizational mentoring strategies designed to enable opportunities and access for women at all levels of leadership led to perceptions of increased commitment, transforming individuals into advocates for the organization (eTable 2, subtheme 3.2, in Supplement 1). The value of mentoring was not specific to women, but effort was needed for women to be afforded the same kind of consistent mentoring opportunities seemingly afforded to men. Likewise, mentoring was perceived as generating better leaders who were capable of leading better teams and driving continuous improvement and better outcomes (eTable 2, subtheme 3.3, in Supplement 1).

Limitations of “plugging in” top-down mentoring programs—matching individuals without creating the necessary conditions—was highlighted, as were benefits of bottom-up engagement and relationship building for long-term outcomes. Mentoring was reported to help organizations and leaders to see their employees, obtaining insights into their personal and workplace needs and accessing highly motivated employees who could benefit the workforce, and creating a cohesive culture and improved organizational outcomes.

#### Raising Women’s Credibility to Enable Internalizing a Leadership Identity

The need for women to navigate, overcome, or correct how they are perceived as leaders was the core reported challenge. Here women needed to negotiate their leadership credibility against a highly challenging triple performance standard, pitting their professional competency, likeability, and leadership performance against one another, with an expectation to uphold all. These high performance standards—proving and justifying their right and credibility to hold leadership positions, assumed not theirs until proven otherwise—were rooted in pervasive beliefs that women have limited credibility as leaders (eTable 2, subtheme 4.1, in Supplement 1). Credibility requirements were perceived in part as a manifestation of male dominance in health care leadership, and the organizational embodiment of ideal leadership, reinforcing the traditionally masculine way as the only credible way of leading. This left women in a situation where their decisions and actions were not considered intrinsically credible, requiring additional strategies for legitimacy (eTable 2, subtheme 4.2, in Supplement 1). When organizational practices and conditions failed to affirm women as leaders and directly address diverse leadership credibility, this affected how different genders interacted and reportedly impacted the credibility of women as leaders.

Overall, the model presented herein—grounded in the collective experience of women in health care leadership—explains that a supportive organizational culture seeking to address gender inequity, promote the necessary conditions for women to internalize and accept their identity as leaders, and in turn enable their career advancement was one that actively removed structural barriers, accepted inclusive approaches to leadership, degendered performance expectations, provided targeted mentoring opportunities, and deemphasized the need to prove leadership credibility. Conceptually, this model demonstrates that the pathway from workforce entry to leadership attainment is highly dependent on an organization’s culture being conducive to women’s credibility as leaders. A supportive culture is built on practices that foster an organization’s credibility, collaboration, and continuous improvement, supporting women and their career advancement. Women’s experiences in the workforce are shown to be influenced by bottom-up and top-down factors (Figure). External and internal conditions could either reinforce or mitigate ingrained societal, gendered expectations and shape organizational culture.

Current organizational leadership has a central role for challenging common misconceptions that are influenced by and that influence the internal cultural discourse underpinning cultural norms, limiting women only to what they can do individually. This involves moving away from ad hoc, isolated, inconsistent applications of gender equity practices and policies that undermine opportunities for advancement and perpetuate an unsupportive culture. It involves embracing practices that demonstrate leadership commitment to organizational change, including supporting early wins and spotlighting positive action.

At the core of this model is an element of judgement whereby the women contemplated their experiences and assigned value to the internal organizational conditions to decide which information aligned with their professional identity as a leader and which information was discordant. Women’s confidence in their leadership ability was constructed through cultural conditions, internal and external cues, and standards (Figure) that affect perceptions of their credibility and internalization of their own leadership identity. Informed by external cues from the organizational environment, women reported learning which of their own individual behaviors garnered merit and should be retained, vs which garnered resistance and should be modified. Strengthening women’s identities as leaders appears possible when organizations broaden acceptance of diverse leadership behaviors and resolve gendered standards of how leadership is enacted internally and externally. Organizations could advance here by meeting the conditions reflected in the 4 outlined elements for long-term, sustainable career outcomes for women across career stages.

## Discussion

This constructivist grounded theory study explored experiences of women in health care leadership related to organizational practices that impacted and advanced their careers. Accounts from 28 women were analyzed, and a model of organizational practices that advance women in leadership was generated. Four interrelated elements were identified that create the necessary conditions for an organizational culture to advance women in health care leadership, including (1) identifying and actively addressing systemic barriers; (2) challenging gendered assumptions and expectations of leadership behaviors; (3) providing mentorship to tangibly shape women’s career opportunities; and (4) determining how these conditions contribute toward raising women’s credibility to enable women to internalize a leadership identity. The interrelationships between elements and how they link to a broader, multilevel, top-down and bottom-up conceptualization of organizational culture underpin this model (Figure).

Our findings suggest that organizations can shift the narrative to develop a credible, collaborative, and continuously improving culture necessary to normalize the desired long-term outcomes. The literature supports this by showing that adaptive and innovative organizations view culture as the most critical component of any change and transformation.^36^ However, simply explaining and mandating the need for culture change is insufficient.^1,2,16^ Here we suggest that health care organizations and leaders pay attention to how leadership-related performance can be conceptualized through a gendered lens that is more sensitive to women’s needs and preferred ways of working and supports diversity in leadership. This is consistent with our findings, which also suggest that organizations with the necessary internal conditions were likely to harness high workforce performance resulting from consistent, committed, and credible leadership, accountable to achieving gender equity in their organization. This begins with understanding the career barriers women face and framing these barriers in a way that can support culture change at a systemic level. Gendered framing that permeates through organizations also appears to harness collective action, with lasting commitment and a responsibility to change.^16,17^

Our study suggests that constructing the purpose of change, through a culture of credibility, collaboration, and continuous improvement, can provide a sense of solidarity with women and reinforce commitment and support for women and their credibility as leaders. This needs to be matched with a shared perception of why and how things ought to be, reflected in organizational expectations of collective conduct toward women on their journey to becoming leaders. As women work, they are exposed to cultural conditions that shape their experiences and judgments of the organization and its efforts toward supporting them and their careers. Identifying and implementing successful organizational practices that build a conducive culture and enhance women’s career opportunities remains challenging,^19,24,36^ contributing to failing interventions, for often unclear reasons.

Our conceptual model, grounded in the collective experience of women leaders, suggests that interventions need not fail if organizations give attention to the practices, processes, and structures that contribute to a culture conducive to intervention success. Meanwhile, it is important to consider the influence of internal conditions as adaptive to new beliefs and attitudes around women’s needs, especially when these are antithetical to the status quo. To do that, organizations need to ultimately consider what culture ought to be, in ways where rules of engagement are separated from the dominant culture.

### Limitations

This study has some limitations. At the time of data collection, the participating health care organization and its workforce were heavily impacted by demands of the COVID-19 pandemic. We acknowledge that work environments are influenced by social, economic, and political conditions that shape the experience of individuals and their expectations from the organizations they work for. Private health services differ from public and community services, as do countries and health care systems and external societal and cultural factors, so this may limit the transferability of our findings to other contexts.^31^ However, participants’ reflections were not limited to their current workplace; reflections included their broad career journey encompassing a variety of health care contexts. Validating and translating our conceptual model into practice will require further research into the role of culture in driving positive outcomes from organizational efforts toward gender equity.

This work is currently being extended to the experiences of women who lead in various settings. Further research will refine our understanding and may elicit additional insights around organizational practices and conditions for addressing gender inequity in leadership and facilitating organizational cultures that support women to advance in health care leadership.

## Conclusions

Through analyzing the experiences of women on their journey to leadership within health care, this qualitative study captures key elements on the role of organizations, including (1) identifying and actively addressing systemic barriers, (2) challenging gendered assumptions and expectations of leadership behaviors, (3) providing mentorship to tangibly shape experiences of career opportunities, and (4) in turn contributing toward raising women’s credibility to enable internalizing a leadership identity. Together with internal and external conditions, these elements are important for organizational workforce culture and underpin our organizational model for advancing women in health care leadership. Practices that resonated most with women and were reported most consistently to enable career advancement were those that were clearly embedded within the cultural fabric of the organization. Organizations could reflect on this model to inform how they can transition from ineffective, isolated interventions to practices and processes that create a conducive culture for women’s career advancement.

## Notes

Supplement 1. **eTable 1.** Interview Guide **eTable 2.** Themes, Subthemes, and Exemplar Quotations

Supplement 2.
Data Sharing Statement
