# Supplementary material for: Experiences of Organizational Practices That Advance Women in Health Care Leadership
Source: JAMA Netw Open. 2023 Mar 20;6(3):e233532. doi: 10.1001/jamanetworkopen.2023.3532 (PMC10028487; doi:10.1001/jamanetworkopen.2023.3532)
Supplement: Supplement 1. — eTable 1. Interview Guide eTable 2. Themes, Subthemes, and Exemplar Quotations [file jamanetwopen-e233532-s001.pdf]

Supplementary Online Content

Mousa M, Garth B, Boyle JA, Riach K, Teede HJ. Experiences of organizational practices that advance women in health care leadership. *JAMA Netw Open*. 2023;6(3):e233532. doi:10.1001/jamanetworkopen.2023.3532

eTable 1. Interview Guide

eTable 2. Themes, Subthemes, and Exemplar Quotations

This supplementary material has been provided by the authors to give readers additional information about their work.

© 2023 Mousa M et al. *JAMA Network Open*.

eTable 1. Interview Guide

ID	Opening Items + Organisational practice and policy (c1)	Subpoints
1	Start by telling me briefly about yourself, your background, and 3 key milestones from your leadership journey.	Self-reflection, tease out organisational activity- the things that make a difference
2	How do you define power? (*preamble: In large organisations, it often appears that key decisions are affected through influence, and influence comes from power.)*	Do you think women in healthcare have power or are they disempowered because of their gender? And why?
3	How would you describe the differences between male and female career trajectories and the impact of that on attaining healthcare leadership?	What gender-based difference do they see in the workforce.
4	How would you describe the orgs responsibility towards you as a woman in health care, and towards other women wanting to advance into leadership?	Define responsibility if required
5	In your org, how does the leadership team approach decision-making? Could you give an example of when you've witnessed gender inclusive leadership?	Tease out if it is top-down command and control leadership approach, or a bottom-up collaborate and consult approach?
**ID**	**Organisational practice and policy (c1)**	**Subpoints**
1	In what ways does the current leadership culture in your immediate work differ from the leadership cultures you've experienced elsewhere?	Looking for leadership accountability, commitment, and visibility
2	In your experience, what did your org have in place to support you and other women pursuing leadership?	Looking for flexible arrangements, practices, policies, enforcement mechanisms, opportunities and resources
3	What was the role of org policy in effecting change for you and for other women in leadership?	Looking for reporting mechanisms, interpretation, structured practice, compliance, and consistency
4	How can orgs ensure that the burden of 'change' doesn't fall on the individual/ avoiding the individual crusade? Give me an example of how an organisation can collectively tackle gender equity issues?	Tease out individual champions and perception of collective effort

© 2023 Mousa M et al. *JAMA Network Open*.

ID	Organisational culture, awareness and engagement (c2)	Subpoints
1	How does the culture of your workplace impact the practices and policies you see on a day to day basis? Can you give me a brief example of where this is modelled in your workplace?	Establish perceived effect of culture on how practice is realized and interdependencies?
2	In your experience, do you think gender bias is an issue in health care culture?	And how does it affect women's career advancement? Has it played out in your experience?
3	What would make the health care workforce become more engaged in a gender inclusive culture?	Tease out awareness and utility of resources to challenge discrimination, inequity, access to support
**ID**	**Organisational support (c3)**	**Subpoints**
1	What should orgs commit as resources to enable women with advancing in their careers?	Looking for internal/ external and industry support, rem and reward,
2	What are some strategies orgs ought to have in place to develop talented women in healthcare? What about acknowledging and celebrating the achievements of women in healthcare?	Tease out identification, early succession planning, mentoring, development, visibility of other women, opportunity, promotion, rem and reward, immaterial support.
3	How can organisations support women from culturally diverse backgrounds with participation in leadership?	Tease out perception of intersectional access to opportunity.
**ID**	**Mentoring and Networks (c4)**	**Subpoints**
1	What are some ways members (leaders, HR, colleagues, DR) within an org can increase tangible support for women's careers? Why would this be important to implement?	Looking for strategies to increase visibility, mentoring, networks, coaching and awareness of impact.
2	In your experience, what are the benefits of participation in a professional network?	Tease out mixed versus female only networking, perceived versus tangible benefits, internal versus external networks
3	In your experience, what are the benefits of being mentored? *(preamble: In this context, formal or informal mentoring from someone from within or outside your area of work- differentiate from coaching if necessary)*	Tease out mentee experiences, learned skills, assoc. to career progression, tangible versus intangible benefits, formal versus informal approaches and key components for success.

© 2023 Mousa M et al. *JAMA Network Open*.

**ID**	**Training and Development (c5)**	**Subpoints**
**1**	Could you give me a short account of how you started to become a leader from a training and development perspective?	Looking for individual and interpersonal capability, content elements, preparation, confidence and readiness
**2**	What do you think are some of the most important skills for people taking on leadership roles in health care to have?	Focus on capabilities, and ongoing effects related to industry needs. Describe what a leader looks like
**3**	What benefits do you think a leadership program focused on women offers the org?	Tease out impact on workforce, impact on patient care.
**ID**	**Closing**	**Subpoints**
**1**	In your opinion, from which positions in your org are you most likely to lose women? And why?	Perception of attrition. Sense of urgency and importance. Ask about career stage as well.
**2**	In your opinion, what is the biggest opportunity for orgs to take today to make an impact for women in health?	Tease out perceived priorities.
**3**	Anything else you would like to add? Closing comments	Offer a copy of summary.

© 2023 Mousa M et al. *JAMA Network Open*.

eTable 2. Themes, Subthemes, and Exemplar Quotations

Example	Exemplar quotation
**Theme 1 Identifying and actively addressing systemic barriers**	
1.1 Organizational attention to barriers specific to women's needs, targeting support	"Something that does worry me…how can I one day maintain this job, which I really want to, if I become a mother, and what are my options? I don't know how comfortable I would feel … having that conversation of what I'm entitled to and what my options would be." PMD
1.2 Providing equitable access for *all* individuals including to family responsibilities and ingrained societal expectations	"It's interesting actually because I don't have children. I think sometimes for women who are in leadership positions and don't have children… there's more of an expectation where you don't have to leave at three o'clock…whereas women in positions that have children, may need to have that work-life balance, so I don't know if we're overtly supportive of that work-life balance in leadership particularly." PN
1.3 Targeting women proactively with opportunities and support	"When you see potential in women, I think it's really important that we recognise talent no matter where they are, communicate that talent, break down the stereotypes. I think having that awareness in our culture is really important." PAH
1.4 Career opportunities - visibility and clarity	"There could be a really easy way for a hospital to do an audit of its practices and see – we should have someone this woman can talk to who looks like them …, and be willing to be accountable to the results of that, and say, well, we clearly don't have enough representation. What are we doing that stop women applying?" PMD
1.5 Career opportunities - the need to recognise and target women proactively	"When I applied for a role there, I was hoping for a leadership role, but I thought, you know, I probably need to start again…When they offered me a leading role here, I was like, oh, my god, I'm going to grab when I was at *****, and here, the way they look after their leaders here, it's just really different. It's totally different." PN
1.6 Credible and implementable policies and practices	"I think we need better promotion practices, not modules at the start of the year on workplace equity that no one takes any notice of." PMD<br>"If you are reported for saying racist, sexist…you will be called in for professional misconduct… It's going to be taken very, very seriously. I think unless people are explicitly told that from a very senior person within a hospital, they assume they can get away with it." PMD
**Theme 2 Challenging gendered assumptions and expectations of leadership behaviours**	
2.1 Social and cultural cues around interactions as leaders	"I think women are disempowered because of gender […] because we're often seen as the more emotive, driven by our hearts […] Sometimes to be successful, you've got to be seen to be powerful and strong, tell people what they need to do... I don't think if there was a male in this role that they would have the same reaction." PN

© 2023 Mousa M et al. *JAMA Network Open*.

2.2 Perceived dichotomy between behavioural benchmarks for men and women	"I've been quite assertive in a meeting, but it's been seen as aggressive, because a man can be aggressive, and it's not seen as aggressive. It's really interesting." PMD
2.3 Descriptive and prescriptive organizational practices - important for counteracting gendered behavioural benchmarks	"I think if you are part of an organisation, even if you're in a relative position of power, and that organisation starts bringing in these changes (i.e. practices to support women) and saying, this is now what we stand for, this is what we're doing. Like it or lump it. If you don't like it, you don't have to work for us, but this is what we're doing. Here's the evidence. This is what we know will happen. We'll get better patient outcomes. Economic productivity improves. Everything is better in terms of workplace culture." PMD<br><br>"The people who are really resistant to change, and really hate it, and don't want to see diversity in terms of power. If they leave, great, you've improved culture already, regardless. In terms of everyone else, you've made a visible display and a structural display of commitment and, hopefully, that emboldens women and other people from diverse backgrounds... to go, okay, I feel safer and I feel more confident. They can keep making change. The little steps that keep bringing women up, and keep bringing from diverse backgrounds up. It's like an escalator, you just keeping doing little steps at a time, and eventually it rolls over into a better environment." PAH
**Theme 3 Mentorship as shaping experiences of career opportunities**	
3.1 Opportunities for the individual and the organisation from being mentored	"I think it's really all about opportunity. Having opportunities to learn from others. You tend to learn a lot from those around you that you don't want to emulate, unfortunately. But it would be nice to be able to have access to some of those really strong leaders in the organisation that you do want to emulate because of what you see and what you hear of them and from them. Some way to foster that culture of learning from each other." PAH
3.2 Structure around mentoring needed to improve effectiveness of opportunities – i.e. access	"I think that's at the goodwill of individuals, and the luck you have finding that mentor who's willing to spend the time. There's nothing formal to help engage that or there's no requirement to do it. In fact, leaders *should* have mentors, really. Where are they learning from? How are they developing, besides being in the trenches every day?" PN
3.3 Organizational mentoring strategies increase commitment and advocacy of individuals	"You have all your leaders learning similar things and similar approaches that, obviously, would align with the organizations ideology … overall concepts of this is the sort of approach we expect our leaders to take, that are open, transparent, whatever approachable. As opposed to leaders finding their own way, and taking their own approach. I think the organisation would get a more consistent group of leaders and benefit from a more consistent approach." PAH

© 2023 Mousa M et al. *JAMA Network Open*.

3.4 Mentoring for generating better leaders	"Mentoring is really important for consistent leadership. I think the stronger your leadership team, the stronger the trust with your floor and your employees. You can't do anything in health care without good employees. Surgeons in private health care won't operate at your hospital unless they've got good patient outcomes. So, it's a cycle of continuous improvement. Good staff that trust good leaders, they enforce good staff, that keep and retain good staff, means good patient care, means more surgeons want to come work for your hospital." PMD
**Theme 4 Raising women's credibility to enable internalizing a leadership identity**	
4.1 Navigating, overcoming or correcting how women are *perceived* as leaders	"whether that's *normally* the traits and attributes you'd put towards a woman…Maybe that's not what people perceive a woman to be. But women in leadership have to be all of those things at times." PAH<br><br>"I think having women in leadership needs to be balanced by getting the right people for the right role at the right time…… there is a risk of not getting the balance right if the right person isn't in the role, and that causes more damage than good." PN
4.2 Organizational embodiment of '*ideal'* leadership reinforcing traditionally masculine ways of leading, as the *only* credible way of leading	"It's definitely about the perception of the power at the top of the tree, and what that top of tree looks like, and perhaps the perception about ***what it takes to be at the top***, and whether that's *normally* the traits and attributes you'd put towards a woman… women in leadership have to be all of those things at times." PAH<br><br>"One thing I learnt on the job back then, 'it' (i.e. proving credibility) was a lot about self-resourcing and being assertive to find the *connections* within the organisation to support your decisions." PMD<br><br>"It's just being strategic about okay, how do I make this person not feel uncomfortable around me, so that they can give me what I need?" PN

Abbreviations: Participant (P), Nursing (N), Medicine (MD), Allied Health (AH)

© 2023 Mousa M et al. *JAMA Network Open*.
