# Supplementary material for: Experiences of Organizational Practices That Advance Women in Health Care Leadership
Source: JAMA Netw Open. 2023 Mar 20;6(3):e233532. doi: 10.1001/jamanetworkopen.2023.3532 (PMC10028487; doi:10.1001/jamanetworkopen.2023.3532)
Supplement: Supplement 2. — Data Sharing Statement [file jamanetwopen-e233532-s002.pdf]

Data Sharing Statement

Mousa. Experiences of Organizational Practices That Advance Women in Health Care Leadership. *JAMA Netw Open*. Published March 20, 2023.
doi:10.1001/jamanetworkopen.2023.3532

Data
Data available: No

Additional Information
Explanation for why data not available: Due to the nature of qualitative research, it is not ethical nor practical to provide the raw data for this study.
